# Optimization of *de novo* transcriptome assembly from high-throughput short read sequencing data improves functional annotation for non-model organisms

**DOI:** 10.1186/1471-2105-13-170

**Published:** 2012-07-18

**Authors:** Berat Z Haznedaroglu, Darryl Reeves, Hamid Rismani-Yazdi, Jordan Peccia

**Affiliations:** 1 Department of Chemical and Environmental Engineering, Yale University, New Haven, CT 06511, USA; 2 Program in Computational Biology and Bioinformatics, Yale University, New Haven, CT 06511, USA; 3 Now at the Department of Chemical Engineering, Massachusetts Institute of Technology, Cambridge, MA, USA

## Abstract

**Background:**

The *k*-mer hash length is a key factor affecting the output of *de novo* transcriptome assembly packages using de Bruijn graph algorithms. Assemblies constructed with varying single *k*-mer choices might result in the loss of unique contiguous sequences (contigs) and relevant biological information. A common solution to this problem is the clustering of single *k*-mer assemblies. Even though annotation is one of the primary goals of a transcriptome assembly, the success of assembly strategies does not consider the impact of *k*-mer selection on the annotation output. This study provides an in-depth *k*-mer selection analysis that is focused on the degree of functional annotation achieved for a non-model organism where no reference genome information is available. Individual *k*-mers and clustered assemblies (CA) were considered using three representative software packages. Pair-wise comparison analyses (between individual *k*-mers and CAs) were produced to reveal missing Kyoto Encyclopedia of Genes and Genomes (KEGG) ortholog identifiers (KOIs), and to determine a strategy that maximizes the recovery of biological information in a *de novo* transcriptome assembly.

**Results:**

Analyses of single *k*-mer assemblies resulted in the generation of various quantities of contigs and functional annotations within the selection window of *k*-mers (*k-*19 to *k-*63). For each *k*-mer in this window, generated assemblies contained certain unique contigs and KOIs that were not present in the other *k*-mer assemblies. Producing a non-redundant CA of *k*-mers 19 to 63 resulted in a more complete functional annotation than any single *k*-mer assembly. However, a fraction of unique annotations remained (~0.19 to 0.27% of total KOIs) in the assemblies of individual *k*-mers (*k-*19 to *k-*63) that were not present in the non-redundant CA. A workflow to recover these unique annotations is presented.

**Conclusions:**

This study demonstrated that different *k*-mer choices result in various quantities of unique contigs per single *k*-mer assembly which affects biological information that is retrievable from the transcriptome. This undesirable effect can be minimized, but not eliminated, with clustering of multi-*k* assemblies with redundancy removal. The complete extraction of biological information in *de novo* transcriptomics studies requires both the production of a CA and efforts to identify unique contigs that are present in individual *k*-mer assemblies but not in the CA.

## Background

Transcriptomic studies using high-throughput sequencing data have enabled researchers to study the global and specific gene expression of many different organisms without the need for a fully sequenced and annotated genome [1,2]. Recently, de Bruijn graph-based [3] software packages such as Oases [4], Trans-ABySS [5], SOAPdenovo [6], and Trinity [7] have been developed to facilitate the transcriptome assembly of massive amounts of short read sequences produced using next generation DNA sequencing technologies. The power and robustness of these packages for forming contiguous sequences (contigs) has been tested, and comparative evaluations on computational resources such as execution time and parallelization, storage, and memory usage have been documented [8-10]. The choice of *k*-mer length (the length parameter defining the sequence overlap between two reads forming a contig) significantly affects the final assembly product [5]. Shorter *k*-mer values might be a better choice in low-coverage studies to prevent the formation of complex overlapping nodes; whereas a larger *k*-mer choice would be more practical for high-coverage sequencing projects [11] to improve assembly accuracy. As an alternative to a single best *k*-mer value selection, multi-*k* value based methods have been adopted to compile different *k*-mer assemblies in order to improve performance, sensitivity, and specificity of the overall *de novo* transcriptome assemblies [2,12]. Multi-*k* value based transcriptome assemblies come along with additional complexities, requiring algorithms to efficiently cluster homologous sequences from each single-*k* assembly and to remove redundant contigs to generate the final non-redundant clustered assembly (CA). Several algorithms such as CD-HIT-EST [13], VMATCH [14], and TGI Clustering tools [15] have been developed to obtain an optimal assembly clustering.

To date, the optimization studies for both single *k*-mer and clustered multi *k*-mer assemblies have largely focused on the length and number of contigs produced as a metric to evaluate the quality of the assembly output. There is, however, a limited understanding of how functional annotation—a primary goal of *de novo *transcriptome analysis—is affected by *k*-mer selection and clustering of multi-*k* assemblies. In this study, we report the significance of *k*-mer selection in the *de novo* assembly and annotation of a non-model eukaryotic organism’s transcriptome with no reference genome information available. We document the variations in uniqueness and the degree of functional annotations obtained under single *k*-mer and multi-*k* clustering methods, and present an assembly strategy to optimize the functional annotation to generate the gene catalogue of a non-model eukaryotic organism. Analysis is performed on Illumina short read sequencing of mRNA transcripts from the microalgae *Neochloris oleoabundans*, a candidate species for the production of microalgae-based biofuels [16,17].

Herein, we also demonstrate that the combination of individual *k*-mer assemblies improves, but does not complete the annotation of all available unique contigs produced in an assembly. A workflow and useful scripts are provided to allow retrieval of additional biological information from contigs that are present in individual *k*-mer assemblies, but not in the clustered *k*-mer assembly.

## Results and discussion

### Sequencing and *de novo* transcriptome assembly

**Figure 1:**
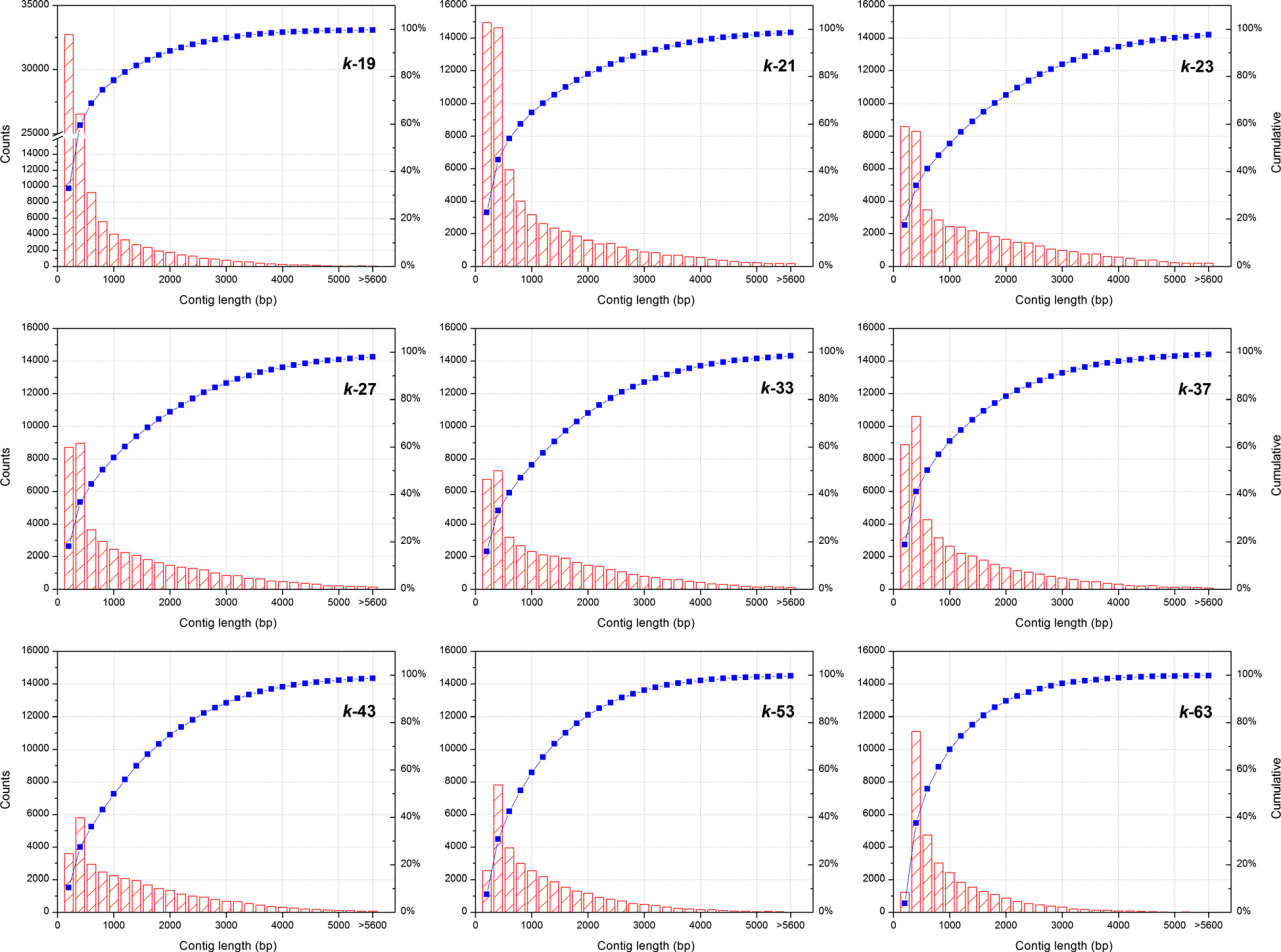
Cumulative contig length frequency distributions for individual assemblies.

Following the removal of short and low-quality reads, the remaining read set was assembled using the combined Velvet and Oases packages [4,11] with single-*k* value selection of odd numbers ranging from 19 to 63. The assembly metrics are provided in Table 1 for the representative *k*-mers: 19, 21, 23, 27, 33, 37, 43, 53, and 63. The number of reads assembled increased gradually from ~18.1 M (for *k*-19 assembly) to ~30.2 M (for *k*-63 assembly), whilst the number of reads mapped was within the range of ~27.8 to 33.3 M for all assemblies. Contig numbers, length distributions, and length-weighted medians (N50 and N90) were comparable among all assemblies, except the *k*-19 assembly (Table 1). The highest number of contigs produced per assembly was 99,438 for the *k*-19 assembly. The contig number steadily decreased to 32,780 as the *k*-mer value increased to 63. Individual contig length and count frequencies are also depicted in Figure 1 for the same representative set of *k*-mers from 19 through 63. These length data (calculated as N50 and N90 in Table 1) and the contig length distribution histograms (Figure 1) demonstrate that a greater contiguity was achieved in mid-range assemblies with *k*-mer selection of 21 to 43 as compared to *k*-19, *k*-53, and *k*-63 assemblies. The average contig length for *k*-21 to *k*-43 assemblies was approximately 1.4 times longer than that of *k*-19, *k*-53, and *k*-63 assemblies. Additionally, the longest average contig length assembled was 1,463 bp in the *k*-23 assembly. 

**Table 1:** Transcriptome sequencing and assembly summary

	***k*-19**	***k-*21**	***k-*23**	***k-*27**	***k-*33**	***k-*37**	***k-*43**	***k*-53**	***k*-63**
**Sequencing**									
Raw sequencing reads					44,568,122				
Read length					99				
**Pre-assembly**									
Reads requiring trimming					29,264,547				
Minimum read length					1				
Lower quartile read length					65				
Median read length					87				
Upper quartile read length					99				
Maximum read length					99				
**Assembly**									
Number of reads assembled	18,097,635	18,481,043	18,018,855	16,878,820	16,918,312	17,188,419	26,970,166	30,231,540	28,058,816
Number of reads mapped	28,449,200	31,893,197	33,199,219	32,903,930	33,395,304	32,523,018	31,939,290	30,267,756	27,808,027
Number of contigs (≥ 100 bp)	98,094	64,000	47,448	46,461	40,965	46,442	34,489	33,344	32,639
Number of contigs (≥ 5,000 bp)	470	1,296	1,587	1, 315	1,025	742	636	253	115
Number of contigs (≥ 8,000 bp)	42	155	215	165	119	72	105	17	22
Average length of contigs	700	1,114	1,463	1,356	1,383	1,115	1,402	1,120	914
Longest contig length	46,754	29,394	16,393	14,115	13,754	13,685	12,571	9,484	12,582
N50	1,594	2,415	2,745	2,624	2,497	2,202	2,349	1,836	1,498
N90	249	470	795	696	733	488	730	515	372

### Effects of *k*-mer selection on mapping, functional annotation, and coverage

**Figure 2:**
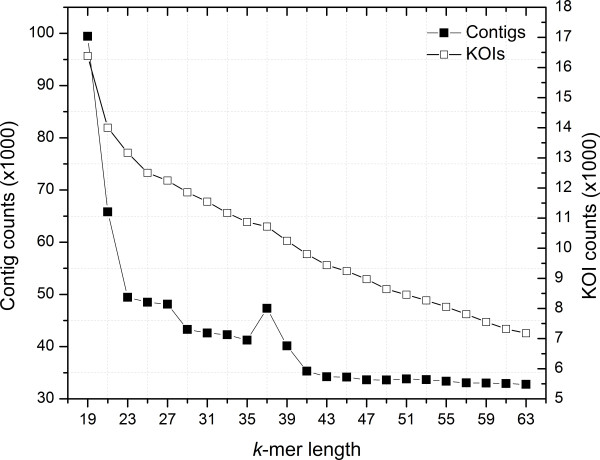
**Total contig and KOI counts for each *k*-mer assembly.**

To compare the differences in attainable functional annotation between each assembly, the contigs originated from single-*k* value assemblies were separately mapped to the KEGG gene and protein families, and the number of unique KEGG Ortholog Identifiers (KOIs) was determined. The number of KOIs identified for a single *k*-mer value reflected the trend previously observed with the contig number (Figure 2). The highest number of KOIs was generated from the *k*-19 assembly, and the number of identified KOIs decreased as the *k*-mer value increased to 63.

**Figure 3:**
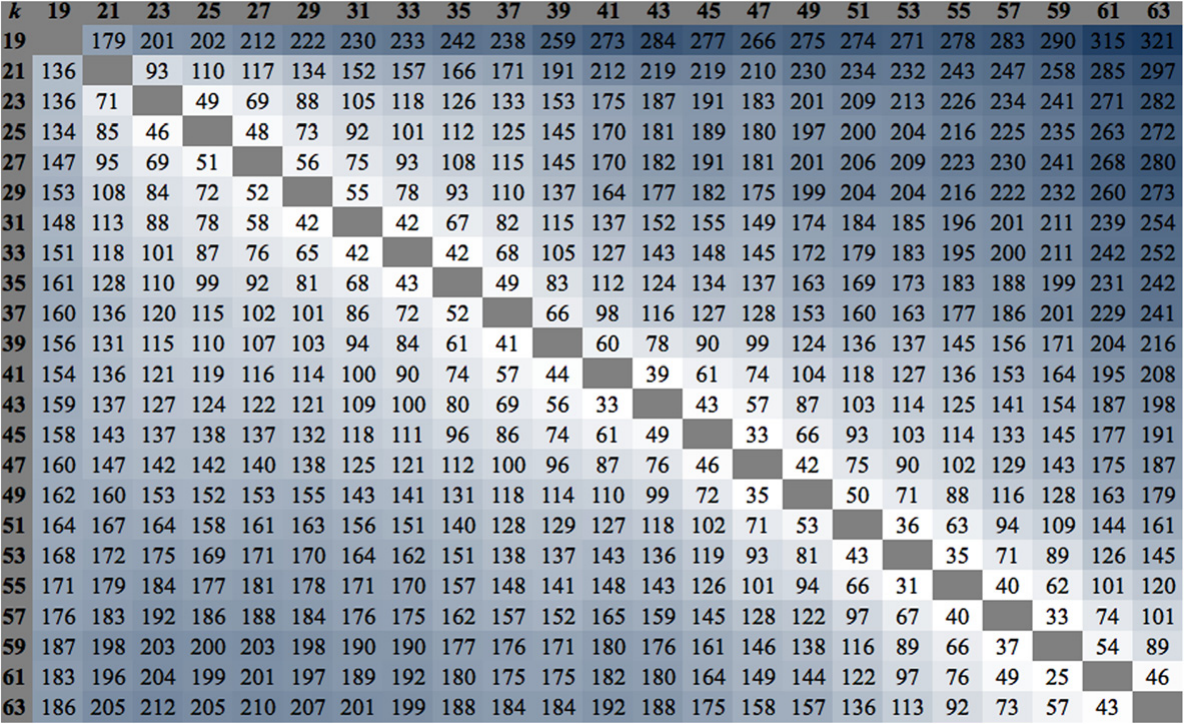
**Comparative matrix of number of unique KOIs missing in each single *k*-mer assembly.** Each value represents the number of unique KOIs (for a specific row *k*-mer value assembly) not identified in the set of column *k*-mer assemblies.

To investigate if each assembly contained a distinct collection of identified genes, the KOIs unique to each *k*-mer assembly were identified and their quantities are presented in Figure 3. This matrix table displays the number of unique KOIs (for a specific row *k-*mer value assembly) not found in the set of column *k*-mer assemblies in a pair-wise comparison. Moving down the *k*-mer column on Figure 3, the *k*-63 assembly resulted in the highest number of unique KOIs that were missing in the other assemblies, followed by *k*-61, *k*-59, *k*-57, and *k*-19. The number of missing KOIs decreased as *k*-mer value increased from 19 to 37 and then increased afterwards.

This analysis has clearly shown that the number of missing KOIs was minimal for mid-range *k*-mers, i.e. from 21 to 41, but it was more prominent for short and long *k*-mer sizes, i.e. 19, 43, and above. The fact that the highest quantities of missing KOIs corresponded to the highest and lowest number of generated contigs, in *k*-19 and *k-*63 assemblies respectively, was not surprising as these two extreme assemblies likely contained more unannotated contigs compared to other single *k*-mer assemblies, where higher accuracy in biological annotation is achieved with optimal mid-range *k*-mer length and ultimately contig length.

**Figure 4:**
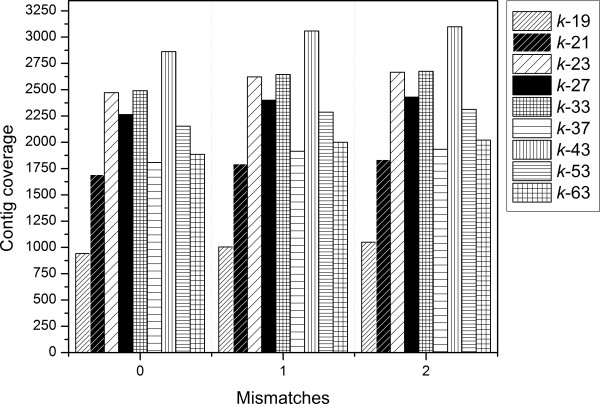
**Contig coverages of representative single *k*-mer assemblies as a function of mismatches allowed by Bowtie mismatches.**

To further characterize the relationship between the single *k*-mer assemblies and the quantities of generated contigs, trimmed reads were mapped to individual *k*-19 to *k*-63 transcriptome assemblies and the fold coverage for each assembly was determined (Figure 4). The results plotted in Figure 4 for the representative *k*-mer set demonstrate that under all mismatch parameters tested (i.e. 0, 1, and 2) the coverage was above 1600× for all *k*-mer values except *k-*19. When one or two mismatches were allowed, more than 2300× coverage was obtained for *k*-mers 23 to 53 except 37. Although lower, the contig coverage for the *k*-19 assembly was still greater than 1000× (Figure 4).

Despite the fact that *k*-mer 19 has resulted in a lower quality assembly in terms of coverage and missing annotations as discussed above, utilization of the k-19 assembly might still have value in annotating the transcriptome. Overall, lower *k*-mer assemblies are more successful in capturing transcripts with lower abundances, but as *k*-mer length increases, transcripts with higher abundances are more likely to be detected. Therefore, all individual assemblies of *k*-19 to *k*-63 were utilized to generate a multi-*k* based CA [11].

### Assembly clustering and optimization

**Figure 5:**
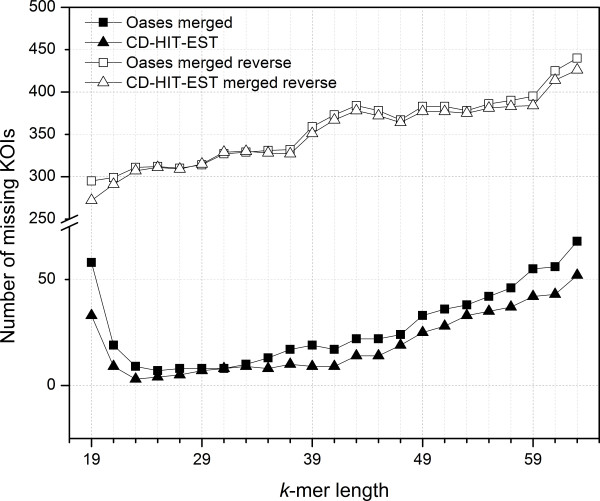
**Number of missing KOIs compared in reverse between clustered assemblies and single *k*-mer assemblies.** Data represent a reverse comparative analysis where the number of KOIs annotated in the CAs, but missing in single *k*-mer assemblies (open triangles for CD-HIT-EST 1.0 and squares for Oases), and the number of KOIs annotated in the single *k*-mer assemblies but missing in the CAs (closed triangles for CD-HIT-EST 1.0 and squares for Oases).

The generation of CA was performed using three different sequence clustering programs: Oases (through its own multi-*k* option), CD-HIT-EST, and VMATCH. The CAs obtained with varying clustering scenarios allowed by these packages were annotated and the number of KOIs present in individual *k*-mer assemblies but not in the CA were determined (Table 2). The best performing package in this regard was the CD-HIT-EST program with 456 missing KOIs in total when a sequence identity threshold parameter of 1.0 was chosen. The Oases package also produced similar results with 635 missing KOIs in total when its multi-*k* option was enabled (Table 2). Furthermore, a reversed comparative analysis was conducted based on the Oases multi-*k* and CD-HIT-EST (1.0) results to determine the number of KOIs annotated in the CA, but not present in individual *k*-mer assemblies (Figure 5). This analysis demonstrated that the CAs resulted in considerably less missing KOIs than did the individual assemblies. In addition the number of missing unique KOIs was ~2.5-7 times less for the CA generated by CD-HIT-EST and ~5-40 times less for CA generated by the Oases multi-*k* compared to the single assemblies of *k*-mers 19 to 63.

**Table 2:** **Number of KOIs present in individual *k*-mer assemblies but missing from the combined assemblies generated with different programs**

***k***	**OASES multi-*k *merged**	**CD-HIT-EST (0.90)**^**1**^	**CD-HIT-EST (0.95)**^**1**^	**CD-HIT-EST (1.0)**^**1**^	**VMATCH**
**19**	58	108	86	33	481
**21**	19	55	41	9	433
**23**	9	37	23	3	402
**25**	7	44	28	4	409
**27**	8	45	29	5	415
**29**	8	53	36	7	420
**31**	8	47	30	8	413
**33**	10	43	27	9	413
**35**	13	51	35	8	422
**37**	17	60	40	10	432
**39**	19	57	38	9	412
**41**	17	59	45	9	412
**43**	22	63	49	14	413
**45**	22	67	52	14	420
**47**	24	76	58	19	426
**49**	33	81	63	25	432
**51**	36	89	73	28	446
**53**	38	95	75	33	447
**55**	42	100	80	35	449
**57**	46	103	83	37	460
**59**	55	113	93	42	473
**61**	56	112	97	43	468
**63**	68	125	110	52	472
**TOTAL**	**635**	**1683**	**1291**	**456**	**9970**

^1^Represents sequence identity.

Nevertheless, Table 2 and Figure 5 collectively indicated that there were still missing KOIs in both CAs and single *k-*mer assemblies. Although the number of missing KOIs in the CA was low compared to the total number of KOIs identified, there was still some relevant biological information lost during this clustering step. This was attributed to the heuristic-based search used in both CD-HIT-EST and Oases to reduce computation time and memory usage, resulting in minor inconsistencies during the removal of redundant sequences.

To further characterize the degree of lost biological information, the missing KOIs identified during the pair-wise comparisons in Figure 5 were subjected to full biological annotation using KEGG BRITE gene and protein families. The missing KOI lists and their full biological annotations are presented as Additional file 1 and Additional file 2 [annotated KOIs missing in single *k*-mer assemblies otherwise present in CAs, corresponding to an average 0.19% of total KOIs, identified with CD-HIT-EST 1.0 scenario (Additional file 1) and 0.27% in Oases multi-*k* (Additional file 2)], and Additional file 3 and Additional file 4 [annotated KOIs missing in CAs otherwise present in single *k*-mer assemblies, corresponding to an average 3.43% of total KOIs, identified with CD-HIT-EST 1.0 scenario (Additional file 3) and 3.48% in Oases multi-*k* (Additional file 4)]. The detailed discussion of lost biological information would be out of the scope of this paper, as its nature and value would differ for each researcher and assembly goal. Nevertheless, a general important interpretation is that there were relevant genes encoding enzymes and proteins (of particular interest for a lipid producing microalgae species with respect to this study) identified as missing in single *k*-mer assemblies but present in CAs (Additional file 1 and Additional file 2) and vice-versa (Additional file 3 and Additional file 4). This suggests that comprehensive annotation should include, in addition to the CA, an interrogation of unique genes in the assemblies of individual *k*-mers from 19 and 63.

### Suggested workflow for optimizing *de novo* transcriptome annotation

Although the generated CA provided the best annotation results, comparison with single *k*-mer assemblies suggested that this approach still results in the loss of some biological information as discussed above. A workflow is presented in Additional file 5, along with several useful scripts, as a guide to improve the annotation of *de novo* assembled transcriptome. The workflow first includes quantifying the number of annotations that could possibly be generated in single *k*-mer assemblies via quick annotation services (such as KAAS) to determine the optimal *k*-mer value range targeted to capture the most comprehensive functional annotation. Next, a clustered assembly should be generated using these *k*-mer values to produce the full set of non-redundant contigs. Finally, pairwise comparisons are performed to identify the unique contigs that are not present in the multi-*k* clustered assembly (otherwise detected in single *k*-mer assemblies). These missing contigs should be incorporated into the final assembly product prior to annotation.

## Conclusions

For the *de novo* transcriptome assembly of non-model organisms from short read sequencing data, de Bruijn graph based algorithms use *k*-mer hash lengths to accommodate transcripts with different sizes. Here, we provide an in-depth analysis of the effects of individual *k*-mer length and multiple *k*-mer assembly methods on transcriptome annotation. Results demonstrate that different *k*-mer choices result in different quantities of unique contigs per single *k*-mer assembly, which in turn impact the amount of biological information that is retrievable from the transcriptome. Although this undesirable effect could be minimized with clustering of multi-*k* assemblies, it is not completely eliminated due to limitations in the heuristic algorithms used in redundancy removal when clustered *k*-mer assemblies are used. We present useful scripts and a workflow to retrieve some of the missing biological information. With high-throughput DNA sequencing methods removing limitations in transcriptome coverage, assembly-based optimization is important for continually improving the completeness of transcriptomes, particularly in non-model organisms for which the reference genome is not available. Taken together, our results provide important guidance on selecting and combining *k*-mer lengths to improve the extraction of biological information from *de novo* transcriptome assemblies.

## Methods

### Algae growth, cDNA sequencing, read trimming, and assembly

*Neochloris oleoabundans* (a Chlorophyceae class green microalgae) was grown in batch cultures under nitrogen stressed and unstressed conditions [17,18]. Total RNA was extracted after 11 days of growth using Rneasy Plant Mini Kit (Qiagen, Germantown, MD). Library preparation was conducted using mRNA-Seq Kit supplied by Illumina (Illumina, Inc., San Diego, CA). Briefly, the mRNA fraction was isolated from total RNA using two rounds of hybridization to Dynaloligo(dT) magnetic beads (Invitrogen, Carlsbad, CA). The mRNA was then fragmented in the presence of divalent cations at 94°C, and subsequently converted into double stranded cDNA following the first- and second-strand cDNA synthesis using random hexamer primers. After polishing the ends of the cDNA using T4 DNA polymerase and Klenow DNA polymerase for 30 min at 20°C, a single adenine base was added to the 3’ ends of cDNA molecules. Illumina mRNA-Seq Kit specific adaptors were then ligated to cDNA 3’ ends. Subsequently, the cDNA was PCR-amplified for 15 cycles and amplicons were purified using the Qiagen PCR purification kit (Qiagen, Germantown, MD). The size and concentration of the cDNA libraries were determined on Agilent 2100 bioanalyzer (Agilent Technologies, Santa Clara, CA). Each cDNA library was loaded onto a lane of the Illumina flow cell and sequenced at the Yale Center for Genome Analysis using a Genome Analyzer IIx and the 99 bp single-read recipe. An additional lane was also used to run sequencing controls. Raw sequencing reads (44,568,122 reads; 99 bp single-ended) of cDNA were analyzed with the FastQC quality control tool (v0.10.0) to evaluate the read sequence quality [19]. Low quality reads with a Phred score value of 13 and less were removed using the SolexaQA software package (v1.1) [20]. After trimming, the FastQC analysis was conducted again to ensure quality measures were met in the remaining reads.

Based on their common application in *de novo *transcriptomic studies using Illumina reads [21,22], the Velvet (v1.2.03) [11] and Oases (v0.2.06) [4] packages were utilized to assemble the high quality reads. In order to investigate the impact of *k*-mer choice on the assembly dynamics, separate assemblies were performed for odd *k*-mer values ranging from 19 to 63 using the “oases_pipeline.py” script provided in the Oases package. The raw sequence data used in this study has been submitted to National Center for Biotechnology Information (NCBI) Short Read Archive (SRA), and are available for public access with accession numbers: SRR391512.1 and SRR391513.1.

### KOI assignment and functional annotation

The resulting contigs from each individual *k*-mer assembly were submitted to the Kyoto Encyclopedia of Genes and Genomes (KEGG) Automatic Annotation Server (KAAS) (v1.6a) [23] for KOI assignment using the default settings with single-directional best hit (SBH) method and databases that included several eukaryotic organisms (including the green microalgae *C. reinhardtii*) [23]. Functional annotation of the KOI assignments was derived from the KEGG BRITE genes and protein families database [24]. Comparison of KOIs for each *k*-mer assembly to KOIs for all other individual *k*-mer assemblies was performed to determine the number of KOIs unique to each *k*-mer assembly. This analysis was generated using a custom built script in the R programming language. This script is provided in Additional file 6.

### Mapping reads to the assembled transcriptome

To understand the relationship between coverage and generated contigs, trimmed reads were mapped against each assembly (*k*-19 to *k*-63) using Bowtie [25] and the contig coverage was estimated. Prior to mapping, any read shorter than the *k*-value of the assembly was removed from the set of trimmed reads. This was done to ensure that reads, which were not used in the assembly, were not mapped to the assembly. Bowtie produced all alignments [5] with 0, 1, and 2 mismatches allowed and utilized the following settings: -a -phred64 -quals -suppress 1,2,4,5,6,7,8 -q -best. Fold coverage was calculated based on the average number of reads mapped per contig in a given *k*-mer assembly. This calculation was performed using a custom designed Python script (provided in Additional file 6).

### Assembly clustering and optimization

Clustered assemblies (CA) were generated from the single *k*-mer assemblies of 19 to 63. Clustering of contigs and redundancy removal were performed using the following three different programs: Oases (by using its incorporated multi-*k* option), CD-HIT-EST (v4.0-2010-04-20) [13], and VMATCH (v2.1.6) [14]. CD-HIT-EST was run with the following parameters: -n 8 -T 4, and 3 different values for the '-c' parameter (0.9, 0.95, 1.0). VMATCH was run with the following parameters: -d -p -l 18 -dbcluster 100 0 -v -nonredundant for vmatch and -allout -pl -dna for mkvtree. Contigs from the combined assemblies were submitted to KAAS for KOI assignment as previously described.

Pair-wise comparison of single *k*-mer assemblies versus CA was performed to determine if unique KOIs existed in specific *k*-mer assemblies but not in the clustered assemblies, and vice versa. To make these comparisons, scripts written in the Python programming language were developed (See Additional file 6).

## Competing interests

The authors declare that they have no competing interests. The software packages considered in these analyses were chosen based on their commonly reported usage in the *de novo* transcriptome assembly literature.

## Authors’ contributions

BZH, DR, and HRY contributed equally to this study. BZH and DR conducted bioinformatics analyses, designed, and prepared the manuscript; HRY conceived and designed the study, and performed the molecular bench work. JP contributed to manuscript preparation and carefully reviewed it. All authors read and approved the final manuscript.

## Authors’ information

Berat Z. Haznedaroglu, Darryl Reeves and Hamid Rismani-Yazdi equal authorship.

## Supplementary Material

Additional file 1 **This spreadsheet contains the list of annotated KOIs missing in single *k*-mer assemblies (provided as separate tabs), but present in the clustered assembly obtained by CD-HIT-EST with 1.0 sequence identity.** Click here for file

Additional file 2 **This spreadsheet contains the list of annotated KOIs missing in single *k*-mer assemblies (provided as separate tabs), but present in the clustered assembly obtained by Oases multi-*k *option.** Click here for file

Additional file 3 **This spreadsheet contains the list of annotated KOIs missing in the clustered assembly obtained by CD-HIT-EST with 1.0 sequence identity, but present in the corresponding single *k*-mer assemblies (provided as separate tabs).** Click here for file

Additional file 4 **This spreadsheet contains the list of annotated KOIs missing in the clustered assembly obtained by Oases multi-*k *option, but present in the corresponding single *k*-mer assemblies (provided as separate tabs).** Click here for file

Additional file 5 **This file provides the reader with a representative workflow to generate optimized *de novo *transcriptome assembly.** Click here for file

Additional file 6 This file contains in-house designed scripts used during the course of the study. Click here for file
