# Supplementary material for: Optimization of de novo transcriptome assembly from high-throughput short read sequencing data improves functional annotation for non-model organisms
Source: BMC Bioinformatics. 2012 Jul 18;13:170. doi: 10.1186/1471-2105-13-170 (PMC3489510; doi:10.1186/1471-2105-13-170)
Supplement: Additional file 5 — This file provides the reader with a representative workflow to generate optimized de novo transcriptome assembly. [file 1471-2105-13-170-S5.ppt]

## Slide 1
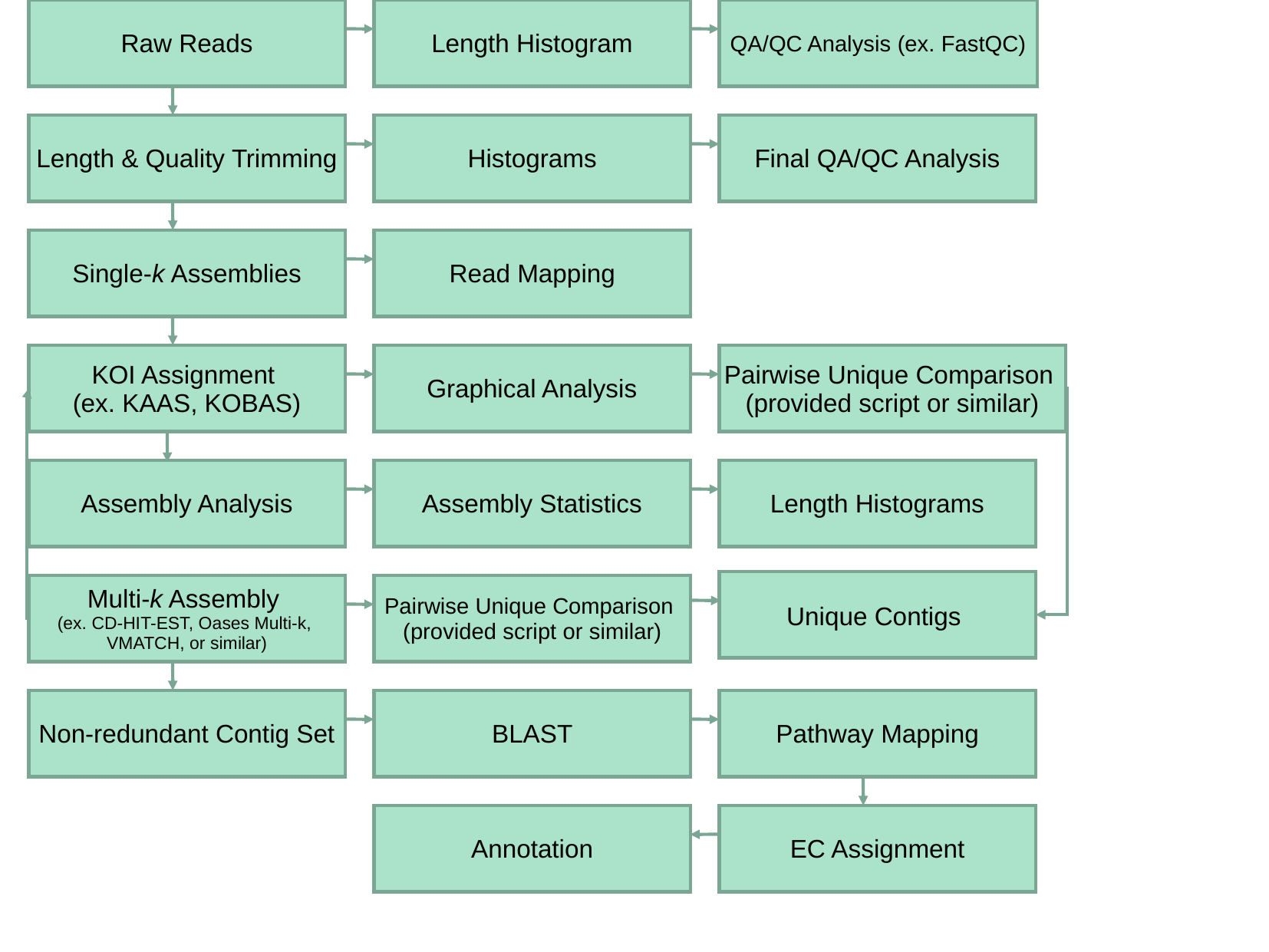

Raw Reads
Length Histogram
QA/QC Analysis (ex. FastQC)
Length & Quality Trimming
Histograms
Final QA/QC Analysis
Single-k Assemblies
Read Mapping
KOI Assignment
(ex. KAAS, KOBAS)
Graphical Analysis
Pairwise Unique Comparison
(provided script or similar)
Assembly Analysis
Assembly Statistics
Length Histograms
Unique Contigs
Multi-k Assembly
(ex. CD-HIT-EST, Oases Multi-k,
VMATCH, or similar)
Pairwise Unique Comparison
(provided script or similar)
Non-redundant Contig Set
BLAST
Pathway Mapping
Annotation
EC Assignment
